# Magneto-optical binding in the near field

**DOI:** 10.1038/s41598-021-00217-6

**Published:** 2021-10-21

**Authors:** Shulamit Edelstein, Antonio García-Martín, Pedro A. Serena, Manuel I. Marqués

**Affiliations:** 1 Instituto de Ciencia de Materiales de Madrid (ICMM-CSIC), Campus de Cantoblanco, 28049 Madrid, Spain; 2 Instituto de Micro y Nanotecnología IMN-CNM, CSIC, CEI UAM+CSIC, Isaac Newton 8, Tres Cantos, 28760 Madrid, Spain; 3 Departamento de Física de Materiales, IFIMAC and Instituto de Física de Materiales “Nicolás Cabrera” Universidad Autónoma de Madrid, 28049 Madrid, Spain

**Keywords:** Magneto-optics, Nanophotonics and plasmonics, Optical manipulation and tweezers

## Abstract

In this paper we show analytically and numerically the formation of a near-field stable optical binding between two identical plasmonic particles, induced by an incident plane wave. The equilibrium binding distance is controlled by the angle between the polarization plane of the incoming field and the dimer axis, for which we have calculated an explicit formula. We have found that the condition to achieve stable binding depends on the particle’s dielectric function and happens near the frequency of the dipole plasmonic resonance. The binding stiffness of this stable attaching interaction is four orders of magnitude larger than the usual far-field optical binding and is formed orthogonal to the propagation direction of the incident beam (transverse binding). The binding distance can be further manipulated considering the magneto-optical effect and an equation relating the desired equilibrium distance with the required external magnetic field is obtained. Finally, the effect induced by the proposed binding method is tested using molecular dynamics simulations. Our study paves the way to achieve complete control of near-field binding forces between plasmonic nanoparticles.

## Introduction

Inter-particle forces produced by light scattering are able to generate optically bound matter where particles are self-arranged in different equilibrium configurations. For the vast majority of stable optical binding configurations, the magnitude of the inter-particle separation is characteristically similar to the incident wavelength.

The first demonstration of optical binding forces dates back to the 80s. Micron size polystyrene particles under light fields were observed to form bound structures orthogonal to the direction of propagation of the incident beam^1^. Later on, longitudinal optical binding was demonstrated where particles in optical trap assumed equilibrium positions along the propagation direction of the incident light field^2,3^.

Within the last 10 years, the experimental difficulties of observing optical binding between nanoparticles have been overcome and optical binding between gold^4^ and silver^5^ nanoparticles has been demonstrated. As with micron-size particles, plasmonic nanoparticles arrange themselves with minimum distance at the order of one wavelength.

Recently, equilibrium separation much smaller than traditional optical binding separations was achieved by using a silver nanowire to construct interferometric optical tweezers^6^. Recent theoretical studies have shown that by introducing surface plasmon polariton interference^7^ or hyperbolic metasurface^8^, the separation between bound nanoparticles can be reduced to less than half of the incident wavelength.

Optical binding between nanoparticles has been studied extensively with the dipole approximation or Coupled Dipole Method^9–15^, with Maxwell stress tensor (MST)^13,16–18^ and from quantum electrodynamical (QED) viewpoint^19–22^. However, many studies have limited the analysis of equilibrium positions to parallel and perpendicular polarization configurations^9,10,12–14,17,21^ and some^9,10,12^ made the “weak interaction approximation” which neglects the effect of multi-scattering and assumes that the dipole associated with the spheres to be due only to the incident field (a good approximation when the polarizabilities are small). As a result, the general consensus is that stable optical binding in the near-field is impossible between two identical nanoparticles.

Optical binding due to the magnetic response of materials has been studied for magneto-dielectric particles^23,24^ and for chiral particles^25^. However, optical binding between particles with magneto-optical response has never been studied. Tuning of optical force and torque by an external magnetic field due to magneto-optical effect has shown to generate novel phenomena such as Stern-Gerlach forces and the existence of a permanent nonreciprocal torque^26^.

Here, we revisit the dimer system considering multi-scattering effects whose importance in optical binding has been demonstrated^27^. By introducing a polarization modulation technique^28^ along with polarization configuration which is neither perpendicular nor parallel to the dimer axis, we demonstrate that it is possible to achieve near-field equilibrium position between two identical plasmonic particles within the Rayleigh regime. Moreover, the equilibrium distance can be controlled to the nanometer scale by the polarization configuration. By analyzing optical binding between magneto-optical particles, we have found that the magneto-optical effect creates a phenomena similar to that of polarization angle and stable near-field equilibrium positions can be tuned by the magnitude of an external magnetic field.

Thus, the manuscript is organized as follows, first we develop the formalism where we show optical binding of identical particles in the near field, then we apply it for a realistic resonant system consisting in two identical silver nanoparticles, and finally we show that this near field binding can be further controlled by means of the magneto-optical effect, by considering in this case InSb nanoparticles. To complete the work we perform an analysis of the seemingly unavoidable azimuth force, and its diminishing upon modulation of the polarization. This can be done either modulating the beam incidence or the polarizability itself e.g. using magneto-optical effects. All these findings are checked against Langevin molecular dynamic simulations.

## System analyzed and the equilibrium condition

In this work, we consider two Rayleigh dipoles with the same polarizability $\hat{\alpha }$, located in $\mathbf{r} _1=(0,0,0)$ and $\mathbf{r} _2=(d,0,0)$ in an otherwise homogeneous and isotropic medium with permittivity $\epsilon _h$ and real refractive index $n_h = \sqrt{\epsilon _h}$. The particles are illuminated with a linearly polarized plane wave $\mathbf{E} _0 (x,y,z)=(E_{0x},E_{0y},0)=(A_{0x},A_{0y},0)e^{i(kz-\omega t)}$, being $k = \frac{n_h\omega }{c}$ the wave number, $\omega$ the angular frequency and $A_{0x}=E_0cos(\theta )$, $A_{0y}=E_0sin(\theta )$ the amplitude components. The total force on the system in the *x*-direction is equal to zero and the force felt by the second dipole is given by^9,10^

1 $$\begin{aligned} F_x =\frac{1}{2}\text {Re}[p_x\partial _xE_x^{*}+p_y\partial _xE_y^{*}] \end{aligned}$$

where $\mathbf{E}$ is the total electric field at the position of the dipole and $\mathbf{p} =\epsilon _0\epsilon _h\hat{\alpha }{} \mathbf{E}$ the induced electric dipole. In this configuration, the total electric field (the sum of the incident field and the field emitted by the other dipole) is given by

2 $$\begin{aligned} E_x=E_{0x}+\frac{k^2}{\epsilon _0\epsilon _h}G_{xx}p_x \nonumber \\ E_y=E_{0y}+\frac{k^2}{\epsilon _0\epsilon _h}G_{yy}p_y \end{aligned}$$

Being $G_{ij}$ the (*i*, *j*) components of the Green dyadic tensor which is dependent on *d*. The force in the *x*-direction is given by

3 $$\begin{aligned} F_x=\frac{k^2}{2\epsilon _0\epsilon _h}[|p_x|^2 \text {Re}(\partial _xG_{xx})+|p_y|^2 \text {Re}(\partial _xG_{yy})] \end{aligned}$$

Usually^9–12^, the equilibrium points considered, at which this force is equal to zero, are given by either $\text {Re}(\partial _xG_{xx})=0$ for $p_y=0$ or $\text {Re}(\partial _xG_{yy})=0$ for $p_x=0$. These equilibrium points are in the medium-far field region where the minimum distance between the particles is approximately one wavelength.

In the present paper, we are going to analyze in detail the equilibrium points located in the near field region, which are given by

4 $$\begin{aligned} \frac{|p_y|^2}{|p_x|^2}=-\frac{\text {Re}(\partial _xG_{xx})}{\text {Re}(\partial _xG_{yy})} \end{aligned}$$

Considering isotropic particles ($\hat{\alpha }=\alpha I$), the electric fields are given by

5 $$\begin{aligned} E_x=\frac{E_{0x}}{(1-k^2 \alpha G_{xx})} \nonumber \\ E_y=\frac{E_{0y}}{(1-k^2 \alpha G_{yy})} \end{aligned}$$

These fields are obtained self-consistently by solving the scattering problem given by Eq. (2), that includes the full interaction between the dipoles. This is called the multi-scattering method, that is at variance with the elemental single scattering approximation where the electric field scattered by each dipole is simply proportional to the scalar product of the external field and the polarizability. The single scattering approximation is expected to fail for near field distances, as the ones reported in this work.

Subsequently, the force in the *x* direction is equal to

6 $$\begin{aligned} F_x=\frac{k^2 \epsilon _0 \epsilon _h|\alpha |^2 E_0^2}{2}\left[ \frac{\cos ^2(\theta )\text {Re}(\partial _xG_{xx})}{|1-k^2\alpha G_{xx}|^2}+\frac{\sin ^2(\theta )\text {Re}(\partial _xG_{yy})}{|1-k^2\alpha G_{yy}|^2}\right] \end{aligned}$$

and the condition in (4) reduces to the explicit expression

7 $$\begin{aligned} \tan ^2(\theta )=-\frac{\text {Re}(\partial _xG_{xx})|1-k^2\alpha G_{yy}|^2}{\text {Re}(\partial _xG_{yy})|1-k^2\alpha G_{xx}|^2} \end{aligned}$$

which gives the required angle of polarization of the incident plane wave for a given equilibrium distance *d*. Notice that in this configuration, $F_{y} \ne 0$ and azimuthal force arises. We will treat this problem in detail in “The azimuthal force” section.

In order to gain a clearer physical insight about the previous results, we consider briefly the near field approximation. In this approach, the components of the Green dyadic tensor are simply given by:

8 $$\begin{aligned} G_{xx}|_{NF} \sim \frac{2}{4\pi k^2 d^3} \nonumber \\ G_{yy}|_{NF} \sim \frac{-1}{4\pi k^2 d^3} \end{aligned}$$

which reduces the expression for the force (6) to

9 $$\begin{aligned} F_x|_{NF} \sim 6 \pi \epsilon _{0} \epsilon _h|\alpha |^2 d^2\left[ \frac{-2\cos ^2(\theta )}{|4\pi d^3 -2\alpha |^2}+\frac{\sin ^2(\theta )}{|4\pi d^3 +\alpha |^2}\right] \end{aligned}$$

Subsequently, we may obtain an explicit equilibrium condition given by

10 $$\begin{aligned} \tan (\theta ) \sim \sqrt{2}\frac{|4\pi d^3 +\alpha |}{|4\pi d^3 -2\alpha |} \end{aligned}$$

valid within the near field region. Note how, for $\theta =0$ ($\theta =\pi /2$) the force is negative (positive) at all distances and there are no equilibrium points in the near field region.

## The silver nanoparticles

We have analyzed a particular case consisting of two silver nanoparticles of 5 nm radius in vacuum illuminated with an external electromagnetic field with intensity $25 \; \upmu \text{W}/\text{nm}^{2}$.

Assuming a small spherical particle of radius r, the polarizability $\alpha$ is given by^29^ $\alpha =(\alpha _0^{-1}-\frac{ik^3}{6\pi })^{-1}$, where $\alpha _0=3V\frac{\epsilon -\epsilon _h}{\epsilon +2\epsilon _h}$, $V = 4\pi r^3/3$ is the particle volume and $\epsilon$ is the relative permittivity of the sphere.

**Figure 1:**
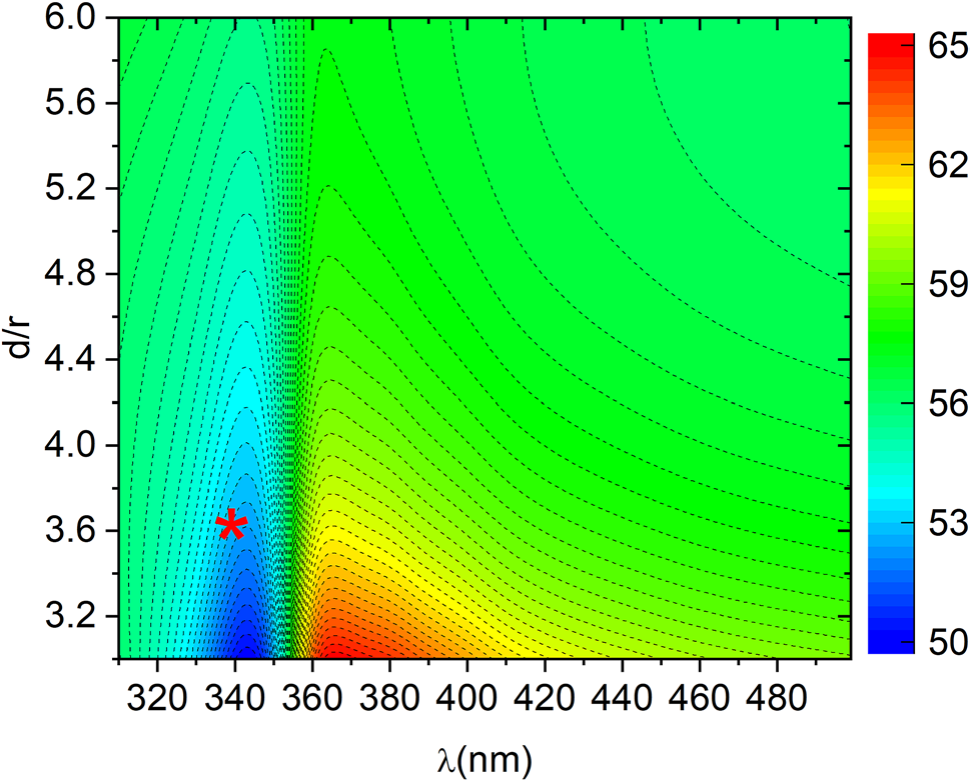
Color map of the polarization angle ($\theta$) needed to obtain an equilibrium binding distance at *d* for each wavelength $\lambda$. Results are for r = 5 nm radius silver nanoparticles.

We have plotted in Fig.1, for each wavelength, the polarization angle required to obtain an equilibrium point at a distance *d* (see Eq. (7)). Note how, for wavelengths close to 340 nm by increasing the angle from roughly 50 to 57°, we change the equilibrium distance from 15 to 30 nm while, for 370 nm, the same equilibrium distances are achieved by decreasing the angle from 65 to 57°.

**Figure 2:**
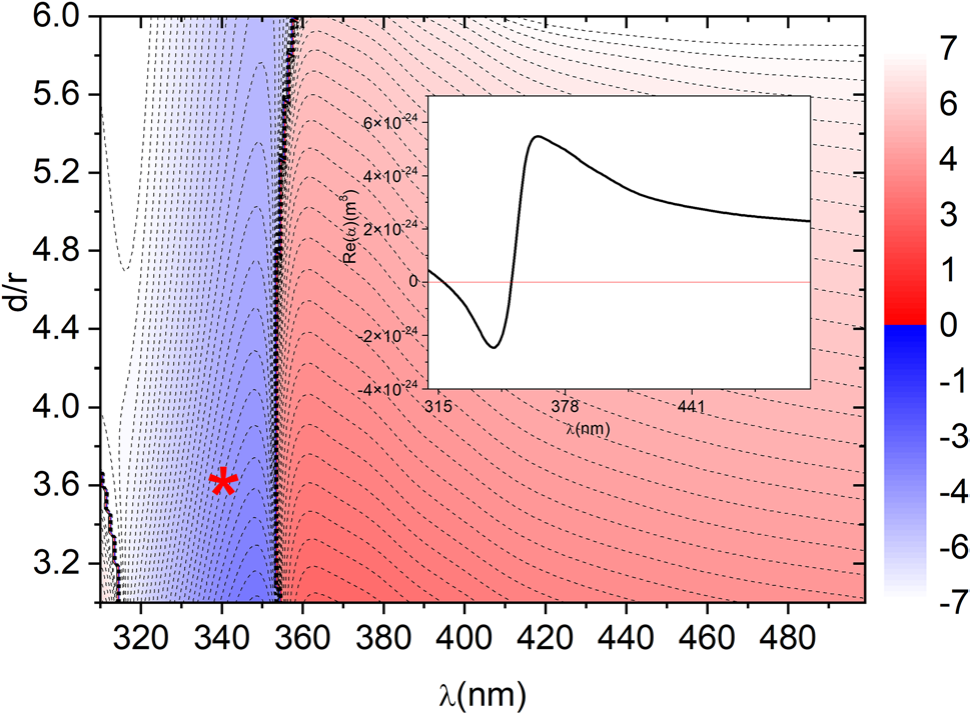
Logarithm color map of the spring constant (in N/m) versus equilibrium distance and wavelength. Negative spring constant (stable equilibrium points) are plotted in blue while positive spring constants (unstable equilibrium points) are plotted in red. Results are for r = 5 nm radius silver nanoparticles illuminated with a plane wave of intensity $25 \; \upmu \text{W}/\text{nm}^{2}$. Inset shows the real part of the particle polarizability versus wavelength. Stable binding is obtained when $\text {Re}(\alpha )<0$.

All values of critical angles are obtained considering the fields given by Eq. (5). However, not all equilibrium distances are stable. The stability regions are shown in Fig. 2, where we plot, at the equilibrium points, the spring constant of the optical force $\kappa =\partial F_x/\partial x$. Stability is represented in blue, while unstable equilibrium points are plotted in red. From Fig. 2, we can infer that stable binding with $\kappa <0$ is achieved for wavelengths with $\text {Re}(\alpha )<0$ (see inset in Fig. 2). $\text {Re}(\alpha )$ is negative near the zone of the resonant excitation of the silver dipole surface plasmon. For gold, $\text {Re}(\alpha )$ is always positive. However, if the particle is embedded in a liquid with a relative permittivity larger than 2.4, then it is possible to get $\text {Re}(\alpha )<0$ also for a 5 nm gold nanoparticle^29^.

**Figure 3:**
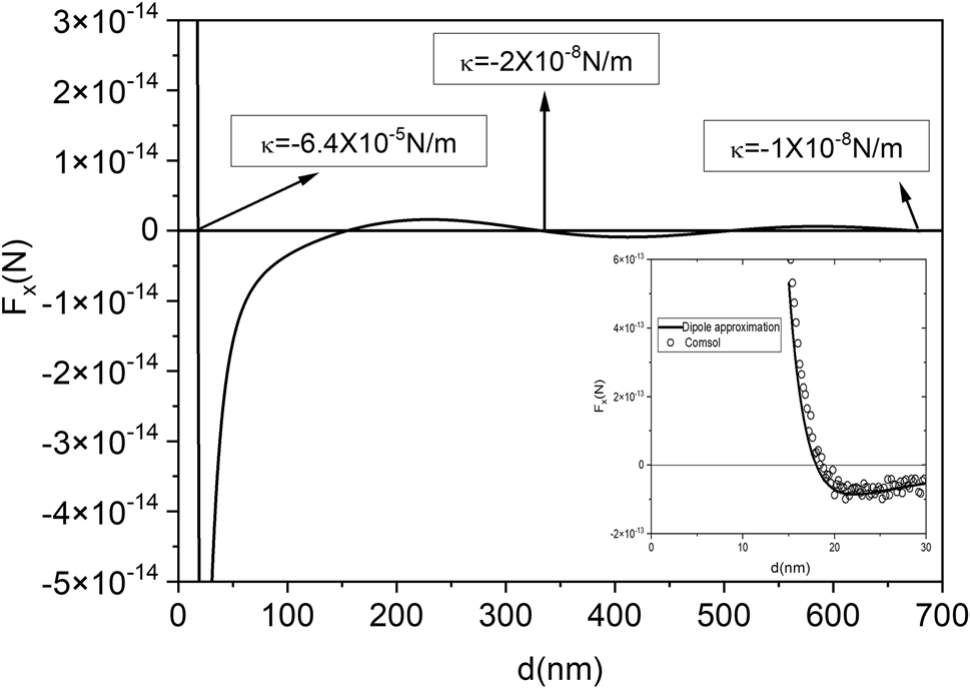
Force versus inter-particle distance for silver nanoparticles of r = 5 nm radius illuminated with a plane wave of intensity $25 \; \upmu \text{W}/\text{nm}^{2}$. The polarization angle and the wavelength considered are marked with a red star in Figs. 1 and 2. The value of the spring constant at three stable equilibrium points (one in the near field and two in the intermediate-far field region) are marked. The inset shows a comparison of the dipole approximation with the finite elements full numerical simulation.

In order to compare the binding effect we have plotted in Fig. 3 the force in the *x* direction versus inter-particle distance for the case of $\lambda =340$ nm and $\theta =52.6^{\circ }$ (marked with a red star in Figs. 1 and 2) which is corresponding to a stable equilibrium distance of 18 nm. The spring constant in the near field is almost four orders of magnitude larger than the far field binding constants located at 333 nm and 677 nm. We have successfully compared these results, obtained within the dipole approximation, with finite elements full numerical simulations using COMSOL Multiphysics (see inset in Fig. 3).

In order to show the validity of the near field approximation, we have compared the critical angle obtained from Eq. (10) (near field) with the result from Eq. (7) (valid at all distances) and we have checked that, for all the angles reported in Fig. 1, the average error using the approximation in (10) is of $3\%$ and the largest error is of $11\%$, that we consider is still sensible for the problem at hand. Note that for a particle with negligible polarizability with respect to the volume, we obtain a zero force angle given by $\tan (\theta )=\sqrt{2}$, which is the value corresponding to static dipoles. At this angle, the force between two static dipoles becomes zero and there is no interaction between them.

By calculating the spring constant $\kappa =\partial _{x}F_x|_{NF}$ at an equilibrium point given by (10), the following condition for a stable configuration is readily obtained:

11 $$\begin{aligned} 8\pi d^6 \text {Re}(\alpha )-2\pi d^3|\alpha |^2-|\alpha |^2\text {Re}(\alpha )<0 \end{aligned}$$

For the silver particles considered, with a radius much smaller than the wavelength, and for binding distances larger than three times the nanoparticle radius, the first term in Eq. (11) dominates and then, the condition to obtain stable binding is reduced to $\text {Re}(\alpha )<0$, which agrees with the result found in Fig. 2.

**Figure 4:**
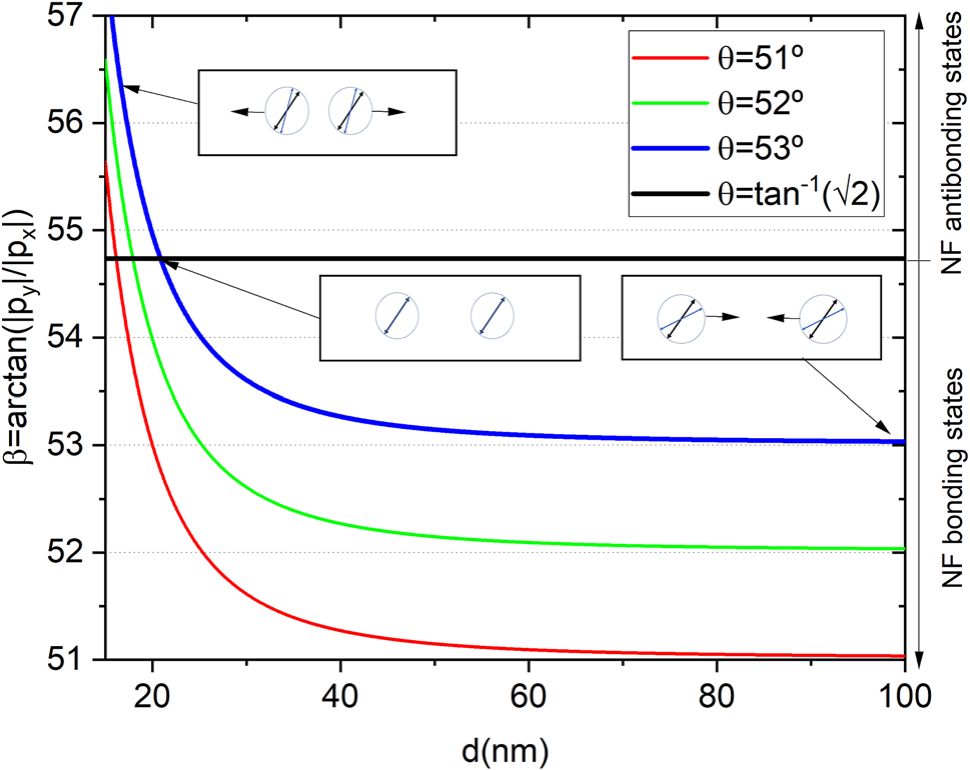
Dipole’s angle with respect to the dimer’s axis ($\beta$) versus interparticle distance for different light polarization angles ($\theta$). Wavelength is set to 340 nm. Black continuous line marks the dipoles angle at which zero bonding force is expected within the near field approximation. In the far field region $\beta \sim \theta < \arctan (\sqrt{2})$ (bonding state) but, as the nanoparticles approach each other, $\beta$ increases. For a particular distance depending on $\theta$, $\beta =\arctan (\sqrt{2})$ and the bonding force vanishes. This is the expected equilibrium binding distance within the near field approximation. As nanoparticles get closer, $\beta > \arctan (\sqrt{2})$ and the anti-bonding repulsion force emerges. Insets show a cartoon of the dipole’s behavior.

The basic mechanism behind the near field binding can be understood considering the quasi-static interaction of the dipoles. A configuration with both dipoles in-phase and lying in the X-axis (dimer’s axis) induces attraction (bonding) while a configuration with both dipoles in-phase and pointing in the Y-axis induces repulsion (anti-bonding)^30^. In the near field approximation, for a dipole’s angle ($\beta =\arctan (|py|/|px|)$) equals to $\arctan (\sqrt{2})$ the force is zero. If the polarization angle ($\theta$) is set to a value slightly smaller than $\arctan (\sqrt{2})$, for large enough interparticle distances, we are going to find $\beta \sim \theta$ and an emerging attractive force (bonding state). However, as the dipoles approach each other, in the near field region, the interaction between the particles is strong enough to noticeable modify the dipole’s angle. Depending on the value of the polarizability, this angle may increase to values larger than $\arctan (\sqrt{2})$ promoting an anti-bonding state or repulsion behavior. In between the two states, we find the stable equilibrium binding distance. This mechanism is explained in detail in Fig. 4.

The equations reported are only valid for small enough particles and interparticle distances large enough to ensure the fulfillment of the dipole approximation. However, the near field binding mechanism also holds for larger particles. As an example, we have found, using exact COMSOL Multiphysics calculations, that near field binding for silver particles with a radius of 40 nm takes place for a distance close to 96 nm for a polarization angle of $72.03^{\circ }$, whereas the dipole approximation predicts a binding distance of 90 nm for the same polarization angle.

## Magneto-optical tuning

For particles with a strong magneto-optical (MO) response it is also possible to tune the binding distance by applying an external magnetic field in the *z* direction $\mathbf{B} _{ext}=(0,0,B_{ext})$. In this case, the polarizability becomes a tensor with out-of-diagonal terms $\alpha _{xy}$ and $-\alpha _{xy}$ inducing a dipole given by

12 $$\begin{aligned} p_x=\epsilon _{0}\epsilon _{h} (\alpha E_{x}- \alpha _{xy} E_{y}) \nonumber \\ p_y=\epsilon _{0}\epsilon _{h}(\alpha E_{y}+\alpha _{xy} E_{x}) \end{aligned}$$

Now the solution to Eq. (2) becomes:

13 $$\begin{aligned} E_x=[E_{0x}(k^2G_{yy}\alpha -1)+E_{0y} k^2 G_{xx} \alpha _{xy}]/\zeta \nonumber \\ E_y=[E_{0y}(k^2G_{xx}\alpha -1)-E_{0x} k^2 G_{yy} \alpha _{xy}]/\zeta \end{aligned}$$

with

14 $$\begin{aligned} \zeta \equiv k^2\alpha (G_{xx}+G_{yy})-1-k^4 G_{yy} G_{xx} (\alpha ^2+\alpha _{xy}^2) \end{aligned}$$

From which we can write the equilibrium condition (4) as:

15 $$\begin{aligned} \frac{|\text {sin}(\theta )(k^2G_{xx}(\alpha ^2+\alpha _{xy}^2)-\alpha )-\text {cos}(\theta ) \alpha _{xy}|^2}{|\text {cos}(\theta )(k^2G_{yy}(\alpha ^2+\alpha _{xy}^2)-\alpha )+\text {sin}(\theta ) \alpha _{xy}|^2}=-\frac{\text {Re}(\partial _xG_{xx})}{\text {Re}(\partial _xG_{yy})} \end{aligned}$$

This condition could be used to find the external magnetic field needed to obtain a desired equilibrium binding distance numerically. However, it is possible to reach an analytic expression for a small enough value of the external magnetic field applied. In that case, we can approach $(\alpha ^2 +\alpha _{xy}^2)\approx \alpha ^2$ and we may consider a linear relationship between $\alpha _{xy}$ and the external magnetic field, given by:

16 $$\begin{aligned} \alpha _{xy}=C_{\alpha }B_{ext} \end{aligned}$$

with

17 $$\begin{aligned} C_{\alpha }\equiv \frac{i\alpha ^2 f}{V\omega (\epsilon -\epsilon _{h})^2} \end{aligned}$$

where *f* is the gyro-magnetic constant given by:

18 $$\begin{aligned} f=\frac{\epsilon _{\infty }\omega _{p}^{2}e}{(\omega +i\Gamma _{f})^{2}m^{*}} \end{aligned}$$

being $\epsilon _{\infty }$ the high frequency dielectric constant, $\omega _{p}$ the plasma frequency, *e* the elementary charge, $\Gamma _{f}$ the free carrier damping constant and $m^{*}$ the effective mass.

Within this approach, the external magnetic field needed to obtain a predefined equilibrium distance is given by:

19 $$\begin{aligned} B_{ext}=\frac{-B_{c}-\sqrt{B_{c}^2-4A_{c}C_{c}}}{2A_{c}} \end{aligned}$$

With

20 $$\begin{aligned} A_{c}&\equiv |C_{\alpha }|^2(\text {cos}^2(\theta )-D_{c}\text {sin}^2(\theta )) \nonumber \\ B_{c}&\equiv -2\text {sin}(\theta )\text {cos}(\theta )\text {Re}(C_{\alpha }^{*}(k^{2}G_{xx}\alpha ^2-\alpha )+D_{c}C_{\alpha }^{*}(k^{2}G_{yy}\alpha ^2-\alpha )) \nonumber \\ C_{c}&\equiv -D_{c}\text {cos}^2(\theta )|k^2G_{yy}\alpha ^2-\alpha |^2+\text {sin}^2(\theta )|k^2G_{xx}\alpha ^2-\alpha |^2 \nonumber \\ D_{c}&\equiv -\frac{\text {Re}(\partial _xG_{xx})}{\text {Re}(\partial _xG_{yy})} \end{aligned}$$

**Figure 5:**
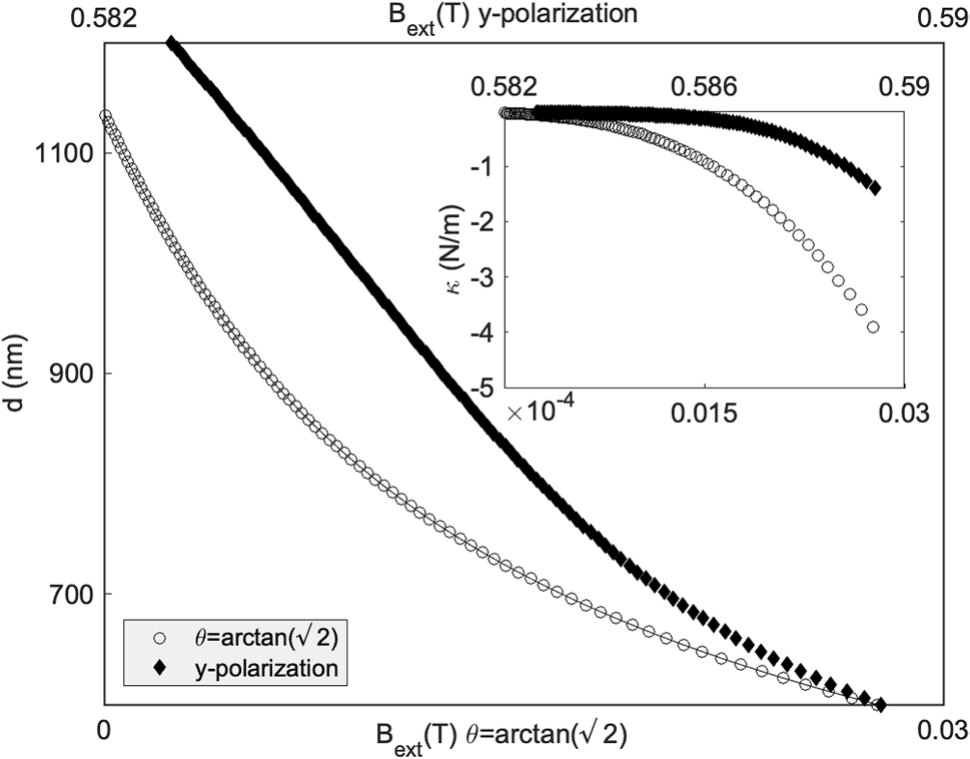
Equilibrium binding distance versus external magnetic field applied for 200 nm radii InSb particles illuminated with a plane wave of wavelength $\lambda =47.97 \; \upmu$m, for *y*-polarization and polarization angle $\theta =\arctan (\sqrt{2})$. Continuous line is the analytic approximation (19) while white points correspond to exact numeric calculation. The inset shows the value of the spring constants at the different equilibrium points for incident beam intensity of $25 \; \upmu \text{W}/\text{nm}^{2}$.

To demonstrate this effect, we consider two n-doped InSb particles, a polar semiconductor that when subjected to an external magnetic field, becomes magneto-optical. The values of the electric and magnetic parameters of this material can be found in reference ^31^. In order to ensure stable binding ($\text {Re}(\alpha ) <0$) we consider a wavelength of $47.9 \; \upmu \text{m}$. At this particular wavelength, and for $\tan (\theta )=\sqrt{2}$, the equilibrium binding distance given by Eq. (7) is at 1136 nm. Next, we apply an external magnetic field ranging from zero to 0.025T and we calculate the induced dipoles given by Eqs. (12) and (13). Then, the binding force is calculated using Eq. (3). With the force, we can calculate numerically the equilibrium distances versus the magnetic field applied and the spring constant at the equilibrium points. It is possible to achieve similar binding effect with *y* polarized incident beam, however larger magnetic fields are required (see Fig. 5).

We have also calculated the necessary external field using the analytic expression (19). Note how, for the range of magnetic fields considered, our prediction matches with the exact result.

## The azimuthal force

The radiation pressure force in the *z*-direction is not considered here as particles are supposed to be lying on a 2D surface either by the existence of a substrate or by the effect of a non-interfering counter-propagating beam of slightly different frequency and same intensity. However, even within this two dimensional approach, there is a force pointing in the azimuth- *y* direction. This azimuthal force pushes the particles away from the equilibrium binding position into a colliding trajectory. The value of this force is given by:

21 $$\begin{aligned} F_y=\frac{1}{2}\mathfrak {R}[p_x\partial _yE_x^{*}+p_y\partial _yE_y^{*}] \end{aligned}$$

and for the configuration considered here, it equals to

22 $$\begin{aligned} F_y=\frac{k^2}{\epsilon _0\epsilon _h}\mathfrak {R}(p_{x}p_{y}^{*})\mathfrak {R}(\partial _{y}G_{xy}) \end{aligned}$$

When a polarization angle of the incoming field $-\theta$ is considered, the incoming field amplitude changes from $(E_{0x},E_{0y},0)$ to $(E_{0x},-E_{0y},0)$. This modification, implies a change on the induced dipole from $(p_x,p_y,0)$ to $(p_x,-p_y,0)$. Similarly, when a negative magnetic field $-B_{ext}$ is applied, the out-of diagonal polarizability term changes sign, causing the same effect on the induced dipole. With these new dipoles, and from the force Eqs. (3), (22), we can check that:

23 $$\begin{aligned} F_x(\theta ,B_{ext})=F_x(-\theta ,-B_{ext}) \nonumber \\ F_y(\theta ,B_{ext})=-F_y(-\theta ,-B_{ext}) \end{aligned}$$

Now, consider that we change the polarization angle and the magnetic field periodically from $(\theta ,B_{ext})$ to $(-\theta ,-B_{ext})$, the average value of the forces is

24 $$\begin{aligned} \langle F_x\rangle =F_x(\theta ,B_{ext})\ne 0 \nonumber \\ \langle F_y\rangle =0 \end{aligned}$$

In conclusion, by changing periodically the sign of the polarization angle and/or the magnetic field, the azimuthal force in the *y* direction disappears while the radial force in the *x* direction remains unchanged. When using larger magnetic fields and *y*-polarized incident beam, only the magnetic field is required to change sign periodically, which implies an easier experimental setup.

## Langevin molecular dynamics testing

In order to test these procedures we have considered a two dimensional Langevin molecular dynamics simulation of two *InSb* spherical particles as the one previously analyzed. The particles are free to move inside a channel of 410 nm diameter made, for instance, of a standing wave optical line trap (SWOLT)^4,28^ or a slot wave-guide^32^.

**Figure 6:**
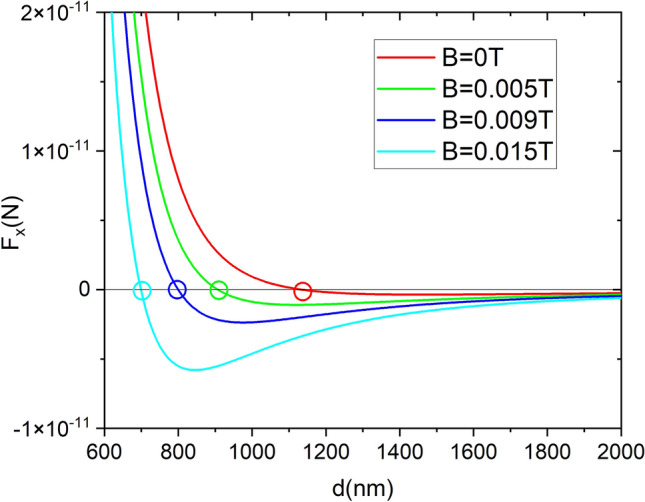
Force versus interparticle distance for several values of the external magnetic field in the case of 200 nm radius InSb particles illuminated with a plane wave of wavelength $\lambda =47.97 \; \upmu$m, polarization angle $\theta =\arctan (\sqrt{2})$ and intensity $25 \; \upmu \text{W}/\text{nm}^{2}$. Empty circles mark the equilibrium distances for each magnetic field.

**Figure 7:**
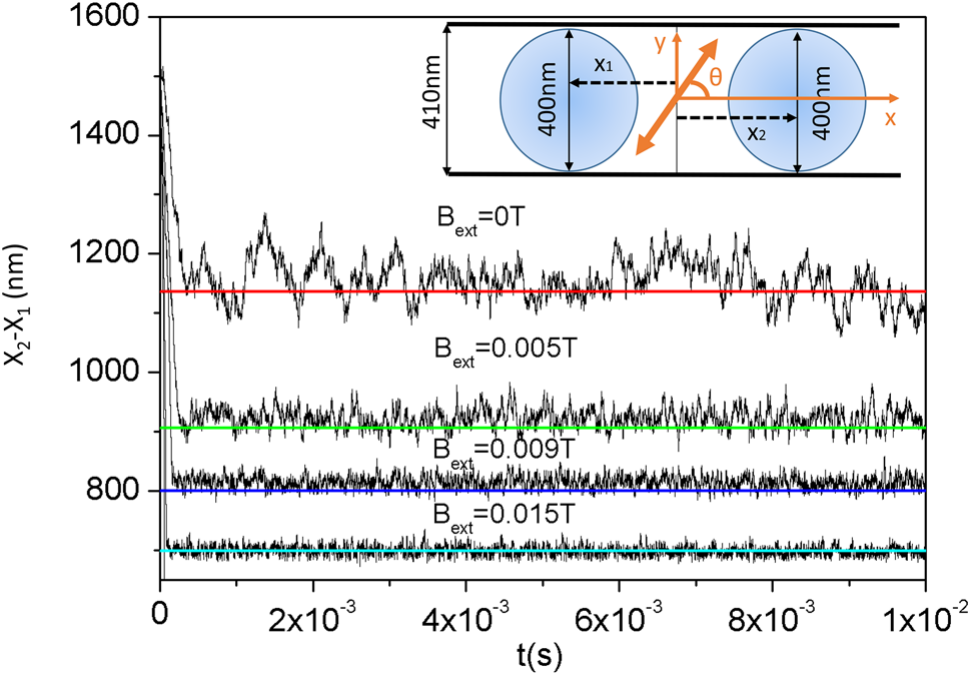
X-Y Langevin molecular dynamics simulation of 200 nm InSb nanoparticles inside a micro-channel with a diameter of $0.41 \; \upmu \text{m}$ illuminated by a plane wave with wavelength $\lambda =47.97 \; \upmu$m, polarization angle $\theta =\arctan (\sqrt{2})$ and intensity $25 \; \upmu \text{W}/\text{nm}^{2}$. Temperature is set to 293K. The simulation shows the particles separation versus time for different values of the external magnetic field. Straight lines correspond to the following expected equilibrium distances, $d=1136$ nm (red), 906 nm (green) 800 nm (blue) 698 nm (cyan), obtained from the analytic expression Eq. (19). Inset shows a sketch of the particles configuration together with the polarization angle and the coordinate system considered.

As initial condition, we consider that the particles are located at a distance of 1500 nm. We consider the same electromagnetic field accounted for in “Magneto-optical tuning” section and then, for a zero external magnetic field, we expect an equilibrium stable binding distance at 1135 nm. Next, we repeat the molecular dynamics simulations using the following external magnetic fields $B_{ext}=0.005 \; \text{T}, 0.009 \; \text{T}, 0.015 \; \text{T}$ which, following Eq. (19), induce the equilibrium distances $d=906 \; \text{nm}, 800\; \text{nm}, 698\; \text{nm}$ respectively (see Fig. 6 for the force versus distance behavior). To decrease the effect of the forces in the *y* direction we consider that the angle of polarization and the external magnetic field change sign with a periodicity of 20 ns. Forces are straightforwardly calculated using the Eqs. (1) and (21). The results for the distance between particles versus time are plotted in Fig. 7 for a temperature of 293 K. Note how the analytic predictions are fulfilled and how the proposed method grants an stable binding distance at the predetermined value.

## Conclusions

In this work, we demonstrate that two plasmonic nanoparticles can form a stable bound dimer even when the separation distance is significantly shorter than the wavelength and as small as three times the radius of the particles. The effect of the near-field binding forces is much larger than the mid-far field forces resulting in enhancement of 4 orders of magnitude of the trap stiffness compared to the common stable optical binding configuration. Moreover, we show that the equilibrium distance can be controlled to the nanometer scale by the angle between the polarization of the incident beam and the separation axis $\theta$.

We also analyze optical binding between two magneto-optical particles and we show that stable near-field binding can be achieved also by applying an external magnetic field. The equilibrium distance can be thus further controlled by the magnitude of the magnetic field. We overcome the azimuthal forces by a periodical modulation of the polarization of the dipoles, either varying the incident beam angle from $\theta$ to $-\theta$, or by using the magneto-optical effect, or both.

## Data Availability

The data that support the findings of this study are available from the corresponding author upon request.
